# The Louder, the Longer: Object Length Perception Is Influenced by Loudness, but Not by Pitch

**DOI:** 10.3390/vision3040057

**Published:** 2019-10-28

**Authors:** Pia Hauck, Heiko Hecht

**Affiliations:** Department of Psychology, Johannes Gutenberg-University Mainz, 55122 Mainz, Germany

**Keywords:** length estimation, object size, impact sound, loudness, pitch, multisensory perception

## Abstract

Sound by itself can be a reliable source of information about an object’s size. For instance, we are able to estimate the size of objects merely on the basis of the sound they make when falling on the floor. Moreover, loudness and pitch are crossmodally linked to size. We investigated if sound has an effect on size estimation even in the presence of visual information, that is if the manipulation of the sound produced by a falling object influences visual length estimation. Participants watched videos of wooden dowels hitting a hard floor and estimated their lengths. Sound was manipulated by (A) increasing (decreasing) overall sound pressure level, (B) swapping sounds among the different dowel lengths, and (C) increasing (decreasing) pitch. Results showed that dowels were perceived to be longer with increased sound pressure level (SPL), but there was no effect of swapped sounds or pitch manipulation. However, in a sound-only-condition, main effects of length and pitch manipulation were found. We conclude that we are able to perceive subtle differences in the acoustic properties of impact sounds and use them to deduce object size when visual cues are eliminated. In contrast, when visual cues are available, only loudness is potent enough to exercise a crossmodal influence on length perception.

## 1. Introduction

What we hear can change what we see, and vice versa. For example, sound localization is altered by vision, as in the ventriloquist effect [1,2,3]. Sounds can also alter our perception of an object’s movement [4,5], its perceived material properties [6], geometric shape [7,8], and hollowness [9]. We know that auditory cues convey information that is crossmodally linked to the size of an object. Evidence for this correspondence was, amongst others, found by means of speeded discrimination tasks in the sense of a pitch-speed compatibility effect: when small objects were paired with a high-pitch tone (as compared to low-pitch tones), discrimination was quicker [10,11]. Similar results were found for matching tasks [12] and two-alternative forced choice (2AFC) paradigms [13,14]. The acoustical parameters of main interest in the mentioned studies were pitch and sound pressure level, and we can derive that small objects are associated with high-pitched and quiet sounds while large objects are linked to low-pitched and loud sounds [15]. This audiovisual connection subliminally influences decision and discrimination processes.

Note that the described effects paired a visual object with more or less unrelated sounds. One might argue that subjects’ attention was captured by the sound because it was particularly salient, and thus constituted unnatural events. We sought to investigate if similar crossmodal effects also arise if the sound source is as natural, that is ecological, as possible.

In the present study, we created an experimental design inspired by Carello, Anderson, and Kunkler-Peck [16] and addressed the question by adopting a modified version of their Gibsonian approach. The ecological approach to perception as shaped by James J. Gibson claims that an event provides information which is directly and integrally connected to a specific source without being cognitively broken down into smaller information units (cf. [17,18,19]). Rather than focusing on perception as mediated by sensation, Gibson believes that information is directly picked up. This process of information pick-up should always be regarded in relation to a person’s natural environment. Thus, rather than employing artificially generated sounds and computerized objects, Carello et al. [16] let participants estimate the length of a wooden dowel, of which they merely heard the sound it made when the experimenter dropped it behind a curtain. Results showed that observers were not only able to discriminate lengths by sound, but their estimates were surprisingly precise for both longer (30–120 cm) and shorter (10–40 cm) dowels (see also [17]). We can conclude from these results that observers are able to extract object length information from sound alone.

Building on this research, we developed a study design to investigate if auditory size perception has a multisensory impact on visual length perception and in how far it can be changed by a more and a less subtle way of sound manipulation. To investigate the nature of potential crossmodal influences, we introduced a brute variation of SPL and a subtler variation by swapping sounds among different dowels. Are image and sound integrated such that the size of a small visual object paired with the sound of a very similar but larger object is overestimated? We presented videos of falling wooden dowels of different lengths and manipulated the impact sounds they made with the floor in three ways. In the main experiment, we exchanged the natural impact sounds among different visual dowel lengths, resulting in images of long dowels combined with the sound of short dowels, and vice versa. These sounds were presented at three sound pressure levels. In two short control experiments, we first added two baseline conditions with only sound and only image, and then altered the pitch of the impact sounds (as opposed to SPL).

If observers are sensitive to the manipulation of exchanging sounds, then a short dowel should be perceived as longer when it is paired with the sound normally produced by a long dowel and vice versa. The larger the difference between the length of the dowel that has originally produced the sound (hereinafter referred to as ‘auditory length’) and the length of the dowel in the image (hereinafter ‘visual length’), the more estimates should deviate from the correct length. We furthermore hypothesized-based on previous findings—that increased SPL would result in longer estimates and decreased SPL in shorter estimates. Finally, we explored if increased pitch would likewise result in shorter and decreased pitch in longer estimates.

## 2. Main Experiment: Manipulation of Sound Level and Swapping Sounds between Dowel Lengths

We presented video clips of short and long dowels falling to the floor and swapped the impact sounds among visual lengths. These stimuli were presented at three sound pressure levels: attenuated, unchanged, and amplified SPL.

### 2.1. Materials and Method

#### 2.1.1. Sample

36 participants took part in the experiment (23 women, 13 men; mean age = 24.8 years, *SD* = 3.6), they were mainly psychology students (2/3 Bachelor’s, 1/3 Master’s students). 16 participants had impaired but corrected-to-normal vision. All were of good hearing (self-reported). The proportion of musicians and non-musicians was approximately balanced (20 answered ‘(rather) yes’, 16 ‘(rather) no’ to the question if they would call themselves a musician).

In accordance with the Declaration of Helsinki, all participants gave their informed written consent, after the topic and potential risks of the study had been explained to them. After the experiment, participants were debriefed about the intention of the experiment. Prior to the study, the Institutional Review Board of the Department of Psychology at the Johannes Gutenberg-University informed us that in accordance with the department’s ethics guidelines no explicit ethics vote of the IRB was necessary for our study, because only harmless visual stimuli were presented, no physiological parameters were measured, and no misleading or wrong information was given to participants.

#### 2.1.2. Apparatus and Stimuli

**Figure 1:**
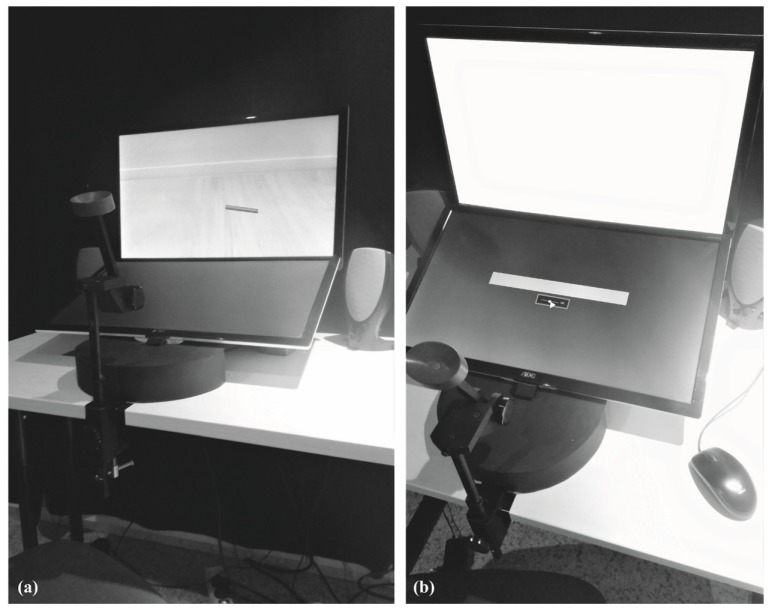
Photos of the experimental setup. (**a**) Stimulus presentation on Monitor A with an image of a fallen dowel (Main Experiment). (**b**) Slider with response bar on Monitor B.

The experiment took place in a laboratory room of the Department of Psychology at the Johannes Gutenberg-University Mainz. Inside a soundproof cabin (210 × 210 × 200 cm), a desk was equipped with a PC (Dell Optiplex 980, Dell Inc., Round Rock, TX, USA), two identical monitors (AOC e2343F2, 23 inch, AOC International, Taibei, Taiwan), and custom PC speakers (harman/kardon, Stamford, CT, USA). One monitor (hereinafter ‘Monitor A’) was mounted in an upright position (forming an angle of approx. 80° with the tabletop) while the other (hereinafter ‘Monitor B’) was tilted by approximately 25° with respect to the tabletop. In front of the desk, a chin rest guaranteed a standardized head position (distances: chin–floor: 102 cm; forehead–Monitor A: 48 cm; ears–loudspeakers: 64 cm). The setup is shown in Figure 1.

**Figure 2:**
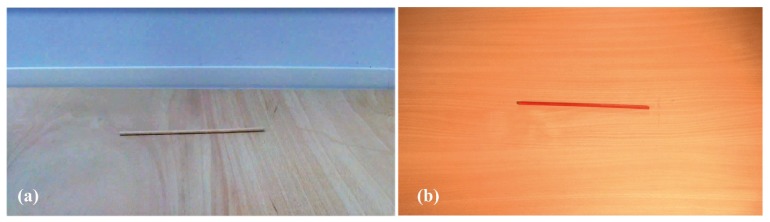
Screenshots of dowel images. (**a**) First video shoot (Main Experiment). (**b**) Second video shoot (Control Experiments).

Visual stimuli: following Carello et al.’s [16] design, we employed beech-wood dowels of seven different lengths as objects. As we wanted to focus on size and at the same time avoid the integration of yet another visual dimension (such as shape mass distribution, material, etc.), we decided to vary only one visual dimension, namely the length of the dowel. All dowels had the same diameter of 6 mm and weighed 2.1 to 8.7 g. The dowels were dropped from a height of 1 m onto a plywood board (120 × 60 × 1 cm) and were filmed with an HD digital video camera (UMA HDDV1, Umazon, Japan) from a side perspective and a distance of 72 cm. Sounds were recorded with the built-in microphone of the camera. The dropping procedure was standardized such that dowels were held with two fingers in their center, laid down onto the edge of a horizontal board, which was attached at a right angle to the wall, and then released to roll over the edge. The video first showed the empty bottom board, and then the dowel falling from above, resulting in a total video length of 2 s (see still image in Figure 2a). There were seven dowel lengths (10–40 cm in 5 cm increments), and each dowel was filmed three times (A, B, C) in order to avoid length recognition on the basis of how the dowel behaved after impact with the surface once it had been dropped (hence called bouncing manner). Bouncing manners varied in dropping speed, in the number and frequency of jumps after the first impact, rolling movements etc. Short dowels jumped more often, higher, and at a higher frequency than long dowels. Examples of videos can be retrieved online (https://bit.ly/2IOxwZ2). Videos were edited with the open-source software Shotcut (version 17.12.03) and formatted as follows: Codec Windows Media Video 8, resolution 1920 × 1080, format yuv420p, frame rate 29.97.

Auditory stimuli: 21 dropping sounds (7 Auditory Length × 3 Bouncing Manner) extracted from the videos described above were employed at three SPLs: original sound level, attenuated by 5 dB and amplified by 5 dB. Original sound level varied by 4.27 dB (LAF_max_) on average between 10 cm- and 40 cm-dowels, so steps of 5 dB seemed appropriate in order to create comparable SPL-differences. The overall maximum sound level was measured with an NTi AL1 sound-level-meter and the volume setting of the replay was chosen so as to create a comfortable maximum sound level for the amplified files (LAF_max_ = 68 dB). Sound files were edited with the open-source softwares Audacity (version 2.2.2) and Shotcut (version 17.12.03) and formatted as follows: Codec Windows Media Audio 2, 2 channels, format fltp, sample rate 48,000 Hz, bit rate 160 kBit/s.

Audiovisual stimuli: 49 new videos (7 Visual Length × 7 Auditory Length) were created. One video of each dowel length was combined with sounds of every dowel length. The use of visual bouncing manners (A, B, C) per visual length was balanced, that is, every video was used at least twice for the combination with seven sounds. The choice of non-corresponding sound (among the three different bouncing manners A, B, C) was made such that it provided the best audiovisual fit, that is the sound most similar to the image in terms of number and frequency of jumps was combined with the image.

#### 2.1.3. Design and Procedure

A full factorial 7 (Visual Length) × 7 (Auditory Length) × 3 (Sound Level) within-subjects design was used. After the experimenter had instructed them, participants sat down in front of the desk and put their chin onto the chin rest. Videos of the dropping dowels were presented on Monitor A followed by a blank screen. With the video offset, a slider appeared on Monitor B, and participants were asked to augment a horizontal grey bar until its length corresponded to the perceived length of the dowel. The bar was augmented by clicking and moving a mouse. The bar could be adjusted to any length between 0 and 40 cm. There were 6 practice trials without feedback and 147 experimental trials with the possibility to take two breaks. We provided no reference or measuring units to scale the responses. The task was to intuitively and quickly reproduce the length just seen. The experiment was programmed with the software Vizard (version 5). In the end, participants were asked to fill in a questionnaire with demographic questions. The experimental session lasted about 40 min.

### 2.2. Results

Due to software deficiencies, a few trials were interrupted during response collection. We decided to replace all responses with unreasonably large reaction times (more than 15 s) by the group mean. Subsequently, outliers were replaced by the group mean, if they lay outside the threefold interquartile range (3 × IQR). Overall, 29 values were replaced, which corresponds to 0.55% of all trials. Between-subjects factors gender, age, educational level, and musicality were not significant for any of the analyses and are therefore not taken into account hereinafter. Data were Greenhouse—Geisser-corrected where necessary. We used the software IBM SPSS statistics 23 for the analyses. Datasets of all three experiments are available as SPSS data files in Supplementary Materials.

#### 2.2.1. Summarily Comparing Judgment Errors of Swapped vs. Unswapped Sound Tracks

A 7 (Visual Length) × 2 (Sound Swap) RM-ANOVA comparing error means of non-manipulated trials (original sound) with error means of all trials with swapped sounds showed no significant main effect of Sound Swap, *F*(1, 35) = 2.37, *p* = 0.133, η^2^ = 0.063.

#### 2.2.2. Analysis of Judged Length as a Function of Visual and Auditory Length and Sound Level

**Figure 3:**
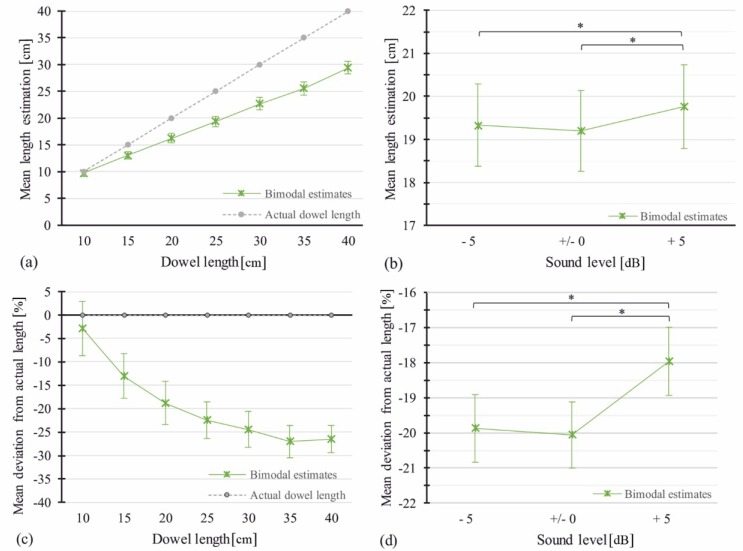
Means of estimated length in the Image-and-Sound condition (bimodal). (**a**) Absolute mean estimates per length. (**b**) Absolute mean estimates per sound level averaged over dowel lengths. (**c**) Error rates: mean percentage deviation from actual dowel length per length. (**d**) Error rates: mean percentage deviation from actual dowel length per sound level. Actual dowel length is shown as a reference. Error bars show ±1 standard error of the mean (SEM). Asterisks indicate significant pairwise comparisons with *p* < 0.01.

A 7 (Visual Length) × 7 (Auditory Length) × 3 (Sound Level) repeated-measures analysis of variance (RM-ANOVA) was carried out for absolute length estimations. For the test values consult Table 1. There was a main effect of Visual Length (see Figure 3a), and a main effect of Sound Level (see Figure 3b). Pairwise comparison of different sound levels revealed a significant difference between attenuated and amplified sound (∆*M* = 0.48, *p* < 0.001) and between normal and amplified sound (∆*M* = 0.51, *p* < 0.001), but no difference between normal and attenuated sound (∆*M* = 0.04, *p* = 0.738, see Figure 3b). The main effect of Auditory Length did not reach significance. Interactions of Visual Length * Auditory Length as well as Visual Length * Sound Level reached significance with differences between Auditory Length and Sound Level being larger for longer dowel lengths (30, 35, 40 cm). Another analogous RM-ANOVA was carried out for percentage deviation from the actual dowel length. Effects were mainly the same as with absolute data, only the interaction Visual Length * Sound Level no longer reached significance. Figure 3c,d show percentage deviation data per visual length and per sound level respectively. Test values can be seen in Table 1.

### 2.3. Discussion

The experiment was designed to answer the question if two different manipulations have an effect on the estimation of dowel length, namely simply varying the sound-pressure level of the falling dowel, and replacing the impact sound by another. Sounds were manipulated such that they were exchanged among visual lengths, that is long dowels were presented with sounds of short dowels and vice versa. In addition, SPL was increased and decreased.

Increasing (decreasing) the SPL led to longer (shorter) length estimates than obtained with the original sound level. The effect was valid for both video types, those with original auditory length and those with non-matching auditory and visual lengths. The more subtle Sound Swap had no comparable effect.

Going into detail regarding the effect of SPL-manipulation, the latter was only significant for differences between the low and the high level, as well as between the normal and the high level, but not between the low and the normal level. We do not have a good explanation for the asymmetry of the effect, but we are in good company. Takeshima and Gyoba [13] likewise found a size-augmenting effect of high-intensity sounds but no compressing effect of low-intensity sounds. The asymmetry might be caused by the loss of sound complexity, which is inherent in recordings. The normal sound of the recording might have been less than what one would expect when faced with the live event. Thus, an augmentation of SPL might be embraced more easily than an attenuation. This is, of course mere speculation. It would have been interesting to compare a larger number of sound levels, for both amplification and attenuation, to test this threshold explanation.

An analysis of relative length judgment errors shows that length estimation was better for shorter than for longer dowels, and even better if sound levels were amplified. Furthermore, the effect of Sound Level was more pronounced for longer than for shorter dowels. We can thus assume that there are detailed acoustic characteristics in the sound helping us to estimate size, which attract more attention and are more easily perceived in the loud condition. In general, length estimation of shorter dowels is apparently easier, and it is less prone to distortion.

Swapping sounds among lengths did not have an effect on length estimation. Sound Swap per se did not reach significance, neither for length estimates nor deviation data. The significant interaction between Auditory and Visual Length shows no clear direction. Thus, the subtler manipulation of compatibility did not produce the hypothesized effect. Participants picked up the overall SLP but not so much the presumably more subtle auditory information when visual and auditory information were conflicting. At this level, merely the visual information made it to the surface. Could the manipulation of sound level have drowned out the subtle auditory variations? Could the videos not have been realistic enough, that is: may the quality of the videos in terms of lighting, perspective, and resolution have not been good enough? The camera position was suboptimal so that perspective distortion might have compromised the visual information. As unlikely as this may be, we wanted to ascertain the auditory and visual information had been transmitted in a suitable way for the purposes of the experiment. We performed two small control experiments to make sure this was the case.

## 3. Control Experiment 1: Multisensory vs. Unimodal Presentation of Swapped Sounds

In a first control experiment, in addition to swapped sounds, baseline conditions were added, which provided only sound or only the visual image. We expected that participants would be able to estimate the relative length of the dowels in all conditions including baseline, assuming that both sound-only and image-only stimuli provide sufficient length information. We also expected estimates on the basis of sound-only stimuli to be affected by pitch manipulation in the same way as image-and-sound videos.

### 3.1. Method

#### 3.1.1. Sample

10 psychology students (6 women, 4 men; mean age: 21.4 years, *SD* = 2.88) took part in the experiment. They had not previously participated. Three participants had impaired but corrected-to-normal vision, all had normal hearing. The proportion of musicians and non-musicians was approximately balanced (6 answered ‘(rather) yes’, 4 ‘(rather) no’ to the question if they would call themselves a musician). All participants gave their informed written consent (see Main Experiment).

#### 3.1.2. Apparatus and Stimuli

The experimental setup was the same as in the Main Experiment.

Visual stimuli: Visual stimuli were designed similarly to those used in the Main Experiment. The videos were shot again for the purpose of several improvements. Dowels were painted red to increase the contrast to the wood-colored ground (60 × 80 cm melamine-coated chipboard, see still image in Figure 2b). Furthermore, the videos were filmed from above in order to reduce perspective distortion (camera elevation 90 cm above the ground at an angle of 60° with respect to horizontal), and a high-quality camera (P2HD 3ccd, Panasonic, Kadoma, Japan) was used for improved overall image quality. Videos were formatted as follows: file type mov, codec H.264/MPEG-4 AVC, resolution 1920 × 1080, format yuc420p, frame rate 25.

Auditory stimuli: 21 dropping sounds (7 Auditory Length × 3 Bouncing Manner) extracted from the videos described above were employed and adjusted to a sound level corresponding to the live sound during the video shoot (approx. LAF_max_ = 80 dB). Sound files were formatted as follows: Codec pcm_s16le, 2 channels, format s16, sample rate 44,100 Hz, bit rate 1665 kBit/s.

Audiovisual stimuli: 49 videos (7 Auditory Length × 7 Visual Length) videos were created and images and sounds were swapped and combined as in the Main Experiment, that is use of images was balanced in terms of bouncing manner, and sounds were matched according to audiovisual fit. In addition, two control conditions were created with silent videos (image-only condition) and videos with only the sound accompanied by a black screen (sound-only condition). Examples of videos can be retrieved online (https://bit.ly/2IOxwZ2).

#### 3.1.3. Design and Procedure

Design and procedure were similar to those of the Main Experiment, with 7 (Auditory Length) × 7 (Visual Length) × 3 (Repetitions) trials in the main block (first). Every trial was repeated three times in order to counteract trial-by-trial variability. A second block of sound-only trials (3 Bouncing Manner × 7 Auditory Length) and a third block of image-only trials (3 Bouncing Manner × 7 Visual Length) were added. Block order was fixed. To rate dowel length, the slide bar on Monitor B could be adjusted to lengths ranging from 0 to 47.7 cm (screen width). Again, the experiment started with 6 practice trials.

### 3.2. Results

Outliers were replaced by the group mean if they lay outside the threefold interquartile range (3 × IQR). This was the case for 6 values, which corresponds to 0.32% of all trials. Between-subjects factors of gender, age, educational level, and musicality were not significant for any of the analyses, they are therefore not taken into account hereinafter. Data were Greenhouse–Geisser-corrected where necessary.

#### 3.2.1. Summarily Comparing Judgment Errors of Swapped vs. Unswapped Sound Tracks as well as Mute vs. Sounding Videos

In order to test for the general influence of swapping sounds, data of all videos with swapped sounds were averaged per length and a 7 (Visual Length) × 2 (Sound Swap) RM-ANOVA was calculated. There was no significant main effect of Sound Swap, *F*(1, 9) = 0.22, *p* = 0.647, η^2^ = 0.02. Furthermore, in order to compare length estimation on the basis of videos with unmanipulated sound with length estimation on the basis of mute videos in the image-only condition, a 7 (Visual Length) × 2 (Sound Presence) RM-ANOVA was carried out. There was no significant main effect of Sound Presence, *F*(1, 9) = 3.63, *p* = 0.089, η^2^ = 0.29, but a tendency towards estimations to be longer with sound than without. Percentage deviation data reveal a tendency towards length estimation of trials with sound being more precise than estimations of mute trials, *F*(1, 9) = 3.84, *p* = 0.082, η^2^ = 0.3.

#### 3.2.2. Image-Only Control Trials: Analysis of Unimodal Visual Estimates

**Figure 4:**
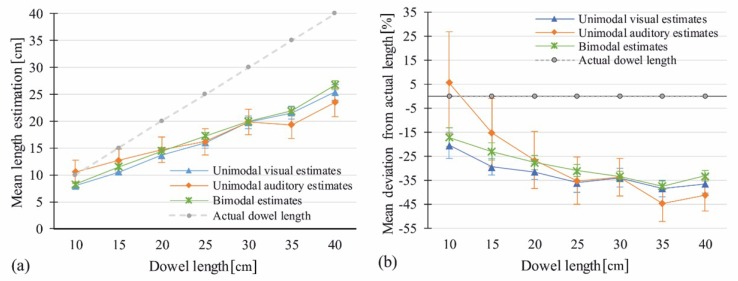
Means of estimated length in the image-only and sound-only condition as compared to bimodal estimates. (**a**) Absolute mean estimates per length. (**b**) Error rates: means of percentage deviation per length. Actual dowel length is shown as a reference. Error bars show ±1 SEM.

A univariate RM-ANOVA was calculated for absolute length estimates in the image-only condition. Estimates were averaged over the three videos with different bouncing behaviors A, B, and C. There was a significant main effect of Visual Length with *F*(2.79, 25.06) = 100.96, *p* < 0.001, η^2^ = 0.92. The analogous RM-ANOVA for percentage deviation data showed the same main effect, *F*(6, 54) = 8.62, *p* < 0.001, η^2^ = 0.49. Figure 4 shows means per length for both data types.

#### 3.2.3. Sound-Only Control Trials: Analysis of Unimodal Auditory Estimates

A univariate RM-ANOVA was calculated for absolute estimates from the sound-only condition. Estimates were averaged over the three videos with different bouncing behaviors A, B, and C. There was a significant main effect of Auditory Length, *F*(2.89, 25.99) = 14.59, *p* < 0.001, η^2^ = 0.62. Percentage deviation data again showed the same main effect, *F*(2.17, 19.54) = 9.35, *p* < 0.001, η^2^ = 0.51. Figure 4 shows means per length for both data types.

#### 3.2.4. Image-and-Sound Trials: Analysis of Judged Length as a Function of Visual and Auditory Length

A 7 (Visual Length) × 7 (Auditory Length) RM-ANOVA was carried out. Data of the three trial repetitions were averaged. There was a main effect of Visual Length, *F*(2.54, 22.87) = 448.37, *p* < 0.001, η^2^ = 0.98, but no main effect of Auditory Length, *F*(6, 54) = 0.62, *p* = 0.71, η^2^ = 0.06. The interaction of Visual Length * Auditory Length did not reach significance, *F*(5, 45) = 1.31, *p* = 0.12, η^2^ = 0.13. The same results were obtained for percentage deviation data, with a main effect of Visual Length, *F*(1.6, 14.34) = 26.42, *p* < 0.001, η^2^ = 0.75, but none of Auditory Length, *F*(6, 54) = 0.37, *p* = 0.893, η^2^ = 0.04, and no interaction of Visual Length * Auditory Length *F*(5.4, 48.6) = 1.1, *p* = 0.37, η^2^ = 0.11. Figure 4 shows means per length for both data types.

### 3.3. Discussion

In Control Experiment 1, we further explored the question of the effect of manipulated sounds. Again, images of various dowel lengths were presented along with sounds from other dowel lengths. This time, control conditions were added, firstly with silent videos, and secondly with sounds accompanied by a black screen. Sound levels of the video replays were adjusted to the respective sound level in the live setting and held constant across all trials.

The results confirm that visual length estimation of a dowel falling on the floor, as watched in a video, is very well possible even when not accompanied by a sound track. Note that lengths were underestimated by 20–40%, which was probably due to the fact that the visual angle subtended by the dowel when watching the video was 25–30% smaller than the visual angle for a would-be observer at the camera position. Analysis of the baseline data in this image-only condition showed that the longer the dowel, the larger the underestimation. Remarkably, analysis of the baseline data in the sound-only condition revealed that the same was true for auditory length estimation. When participants merely heard the dowel fall, they could appropriately estimate its length, but with the same underestimation as mentioned above for image-only trials. That is, the longer the dowel that had produced the impact sound, the longer the participants’ length estimates. Regarding percentage deviation, underestimation was, again, larger the longer the dowel. However, the observers’ ability to hear size changes from sound was not strong or robust enough to be factored into the multisensory length estimates. As in the Main Experiment, there was no effect of Auditory Length for image-and-sound stimuli, and no interaction with Visual Length. At this point, we must consider that insufficient audiovisual fit of the newly created videos might have been a reason for the missing effect. Possibly, participants were irritated by the intermodal asynchronies so that sound was not perceived as belonging to the dowel in the image. However, most participants reported that they did not notice that sounds had been swapped. Thus, such irritations would have factored into the estimates unconsciously.

The fact that mere auditory perception of the impact sound of a falling dowel allowed the observers to estimate its length, demonstrates that auditory information can be appropriately processed, as long as it is the only (or main) source of information. However, this information appears to be under-utilized in the presence of visual information about dowel length. However, note the tendency towards a positive contribution of the auditory modality when the video was supplemented with (correct) sound. Taken together, these results indicate that sound does alter length perception, not only in terms of manipulated loudness.

## 4. Control Experiment 2: Manipulation of Pitch

Thus far, we have manipulated the auditory stimulus by altering loudness (via SPL variation) and subtle cues (via swapping sound tracks). In our second control experiment, we have manipulated pitch, given that it is the remaining salient dimension of sound.

### 4.1. Method

#### 4.1.1. Sample

10 different psychology students (7 women, 3 men; mean age: 24 years, *SD* = 4.6) took part in the experiment. 4 had impaired but corrected-to-normal vision. One person reported a subjective hearing impairment, but as ratings did not notably differ from the rest of the sample, the data were not excluded. The proportion of musicians and non-musicians was balanced (5 ‘(rather) yes’, 5 ‘(rather) no’). All participants gave their informed written consent (see Main Experiment).

#### 4.1.2. Apparatus and Stimuli

The experimental setup was the same as in the Main Experiment and Control Experiment 1.

Visual stimuli: Visual stimuli were the same as in Control Experiment 1.

Auditory stimuli: The same 21 dropping sounds as in Control Experiment 1 were used. These sounds were manipulated in terms of pitch (−10%, −5%, ±0, +5%, +10% (Hz)) with the help of the video editing software Shotcut. Steps of pitch increase and decrease were chosen such that differences were clearly noticeable, but still realistic for the naïve listener. 

Audiovisual stimuli: Other than before, the sound tracks were not swapped among lengths, but the videos of each dowel length were combined with the five pitch-manipulated sound tracks of the related lengths. That is, this time all bimodal trials contained images and sounds matching in length. Again, image-only and sound-only control conditions were added. Examples of videos can be retrieved online (https://bit.ly/2IOxwZ2).

#### 4.1.3. Design and Procedure

Design and procedure were identical to those of Control Experiment 1, with the exception that the swapping of sounds was replaced with a five-step pitch manipulation. This resulted in 105 trials (7 Visual Length × 5 Pitch × 3 repetitions) in the first block (image-and-sound), 35 trials (7 Auditory Length × 5 Pitch) in block 2 (sound-only), and 21 trials (7 Visual Length × 3 Bouncing Manner) in block 3 (image-only).

### 4.2. Results

Outliers were replaced by the group mean, if they lay outside the threefold interquartile range (3 × IQR). This was the case for five values (0.31% of all trials). Between-subjects factors gender, age, educational level, and musicality were not significant for all analyses and are therefore not taken into account hereinafter. Data were Greenhouse–Geisser-corrected where necessary.

#### 4.2.1. Summarily Comparing Judgment Errors of Changed vs. Unchanged Pitch as well as Mute vs. Sounding Videos

In order to test for a general difference between videos with changed pitch and videos with original pitch, data of all videos with manipulated pitch were averaged per length and a 7 (Visual Length) × 2 (Pitch Change) RM-ANOVA was calculated. There was no significant main effect of Pitch Change [absolute data: *F*(1, 9) = 0.48, *p* = 0.51, η^2^ = 0.05, deviation data: *F*(1, 9) = 0.02, *p* = 0.89, η^2^ < 0.01]. Furthermore, as in Control Experiment 1, a 7 (Visual Length) x 2 (Sound Presence) RM-ANOVA was carried out on the basis of image-and-sound videos with unmanipulated sound vs. mute videos in the image-only condition. There was no significant main effect of Sound Presence (absolute data: *F*(1, 9) = 0.03, *p* = 0.871, η^2^ < 0.01; deviation data: *F*(1, 9) = 0, *p* = 0.958, η^2^ < 0.01).

#### 4.2.2. Image-Only Control Trials: Analysis of Unimodal Visual Estimates

**Figure 5:**
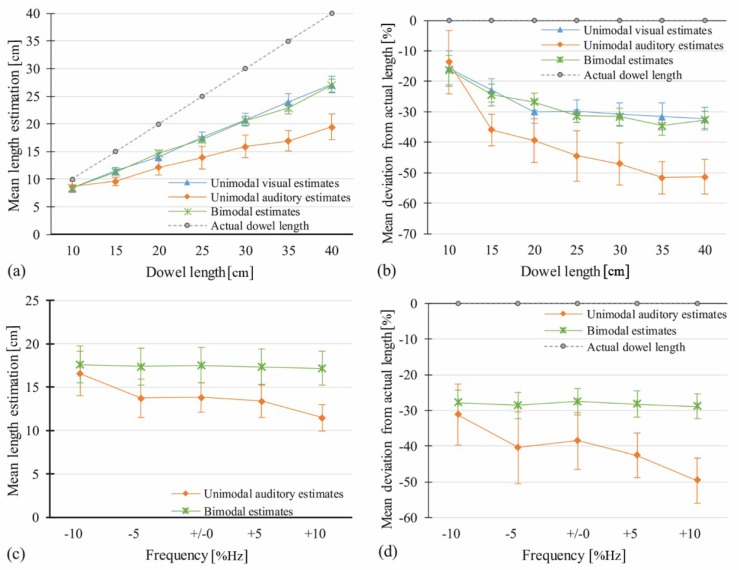
Means of estimated length in the image-only and sound-only condition as compared to bimodal estimates. (**a**) Absolute mean estimates per length. (**b**) Error rates: means of percentage deviation per length. (**c**) Absolute mean estimates per degree of pitch manipulation. (**d**) Error rates: mean percentage deviation per degree of pitch manipulation. Actual dowel length is shown as a reference. Error bars show ±1 SEM.

The same analyses as in Control Experiment 1 were carried out. For the absolute estimates there was a significant main effect of Visual Length, *F*(2.26, 20.31) = 122.68, *p* < 0.001, η^2^ = 0.93 (see Figure 5a). Percentage deviation data showed the same main effect, *F*(2.14, 19.24) = 6.18, *p* = 0.008, η^2^ = 0.41 (see Figure 5b). Figure 5a,b allow the comparison of absolute estimates with the actual dowel length.

#### 4.2.3. Sound-Only Control Trials: Analysis of Unimodal Auditory Estimates

For absolute estimates of the sound-only condition, a 7 (Auditory Length) × 5 (Pitch) RM-ANOVA was carried out. There were both main effects of Auditory Length, *F*(2.67, 24.05) = 30.83, *p* < 0.001, η^2^ = 0.77, and Pitch, *F*(4, 36) = 7.61, *p* < 0.001, η^2^ = 0.46, as well as a significant interaction Auditory Length * Pitch, *F*(24, 216) = 2.01, *p* = 0.005, η^2^ = 0.18. Pitch differences were larger for longer dowel lengths (30, 35, 40 cm). Figure 5 shows mean estimates per length (a) and mean estimates per degree of pitch manipulation (c). The same effects were found for percentage deviation data (Auditory Length: *F*(2.8, 25.32) = 20.12, *p* < 0.001 η^2^ = 0.69, see Figure 5b; Pitch: *F*(4, 36) = 5.25, *p* = 0.002, η^2^ = 0.37, see Figure 5d; Auditory Length*Pitch: *F*(24, 216) = 2.29, *p* = 0.001, η^2^ = 0.2).

#### 4.2.4. Image-and-Sound Trials: Analysis of Judged Length as a Function of Visual Length and Pitch 

A 7 (Visual Length) × 5 (Pitch) RM-ANOVA was calculated for the absolute estimates, and revealed a main effect of Visual Length, *F*(1.32, 11.87) = 205.09, *p* < 0.001, η^2^ = 0.96, but no main effect of Pitch, *F*(4, 36) = 0.82, *p* = 0.52, η^2^ = 0.08. There was a tendency towards an interaction of Visual Length*Pitch, *F*(24, 216) = 1.55, *p* = 0.055, η^2^ = 0.147, with long dowels being estimated slightly longer with decreased pitch. Similar results were obtained for the analysis of percentage deviation data (Visual Length: *F*(1.28, 11.55) = 9.67, *p* = 0.007, η^2^ = 0.52; Pitch: *F*(4, 36) = 0.58, *p* = 0.68, η^2^ = 0.06; Visual Length*Pitch: *F*(24, 216) = 1.64, *p* = 0.035, η^2^ = 0.154). Note that interaction between Visual Length*Pitch did reach significance in the relative error data.

### 4.3. Discussion

As the Main Experiment showed an impact of SPL manipulation, but the effect of sound swapping was not significant neither in the Main Experiment nor in Control Experiment 1, we wanted to test in a second control experiment if an artificial manipulation of pitch to a well noticeable extent would show an effect, just as a well noticeable SPL manipulation did. We expected that increased pitch would result in shorter estimations and decreased pitch in longer estimations.

Again, results show that length estimates are quite accurate if based on visual information alone or on auditory information alone. This confirms the results of Control Experiment 1. There was no influence of altered pitch on length estimation of the multisensory stimulus. However, when confronted with the sound only, participants gave longer estimates when they heard sounds with decreased pitch, and shorter estimates for sounds with increased pitch. This effect is especially pronounced for longer dowel lengths. Percentage deviation data confirmed that there was less length underestimation when pitch was decreased and stronger underestimation when pitch was increased. This result confirms that sound can carry size perception of objects hitting the ground, but it is not important enough to create a multisensory distortion of size perception in settings like ours.

## 5. General Discussion

The aim of the present study was to examine the crossmodal influence of auditory cues on visual size perception of objects. In one main and two control experiments, videos of wooden dowels of different lengths falling to the ground were shown with original or manipulated impact sounds. In the Main Experiment, sound was swapped among lengths, that is visual events of shorter dowels were combined with impact sounds of longer dowels and vice versa and presented at three different loudness levels. In the first control experiment, sounds were again exchanged among lengths, and baseline conditions were added. In the second control experiment, sounds were manipulated in terms of pitch.

The results show that auditory impact cues presented in isolation provide sufficient information to estimate the size of dropping objects. This is consistent with the current state of research on ecological perception (cf. [14,16,17,20]). Furthermore, the results provide evidence that both SPL and pitch manipulations of the sound do influence size estimation in a solely auditory setting. This finding is in line with research on crossmodal associations of pitch and size, as apparent in speeded discrimination tasks [10,11]. In a multimodal setting (vision and sound), manipulation of SPL does exert a multisensory effect, that is a significant change in length estimation. This is consistent with findings by Takeshima and Gyoba [13] who tested in a 2AFC-paradigm if objects are more likely to be identified as bigger than others, when presented together with a loud noise, as opposed to a low noise or silence, which was indeed the case. However, Control Experiment 2 showed that the effect of pitch manipulation vanishes as soon as the sound is presented together with a visual stimulus. The readily available pitch information is ignored, if not actively suppressed in the presence of visual information.

Thus, the loudness dimension of sound-unlike pitch-appears to be a reliable and relatively salient indicator of object size, such that the crossmodal correspondence between loud sounds and large objects, and low sounds and small objects, influences visual perception. One conceivable way the perceptual system accomplishes this effect is an unconscious deduction of the object’s weight, which is then factored into the length estimate. Here, the auditory variant of the size-weight illusion [21,22] comes into play, which can be taken as an association of louder noises with heavier objects, which in turn are perceived as bigger. Note that size-pitch-correspondences and weight-pitch-correspondences (cf. [23,24]), as well as size-loudness correspondences [13,25] well documented. Our findings show, that when paired with a salient visual stimulus, loudness carries much more impact than does pitch. In our stimuli, dowel length was linearly related to dowel weight, thus our experiments also demonstrate a loudness–weight correspondence, which has been missing in the documented literature.

Let us now consider deviation data. Loudness as an important information transmitter becomes even more salient here. Length estimates of dowels presented together with an amplified sound were better, that is closer to the actual dowel length, than those presented with normal and attenuated sound. Overall, error rates for both multimodal and unimodal stimuli were smaller as the dowel got shorter, which is in line with Carello et al.’s [16] results. Also, the main effects for loudness (multimodal stimuli) and pitch (unimodal stimuli) were more pronounced for longer dowel lengths, thus the estimation of longer dowels appears to be more vulnerable. We suppose that estimates for shorter dowels are more precise and more robust due to increased familiarity, e.g., with the sound of a pencil falling on the floor. Furthermore, results show that sound per se helps us to estimate length, as error rates were smaller for multimodal than for unimodal visual trials, that is accuracy of length perception is improved by sound (cf. [26]).

Coming back to our hypothesized effects, a possible way to explain the absence of effects for the subtler manipulation of swapping sounds is to merely regard visual perception as the stronger sense when it comes to an interplay with more complex auditory stimuli. This simple dominance idea is supported by research on the ventriloquism effect (e.g., [3,27]), as well as by the so-called Colavita visual dominance effect [28], which showed up not only in experiments with lights and tones, but also with more emotional stimuli [29]. Findings by Tsay [30] show that sight overrules sound even in a setting where auditory cues are obviously more important and thus gain more attention than visual cues, here during music performance. However, our results point to a more refined role of crossmodal negotiation that takes advantage of the information in a satisficing way. When present and appropriate, auditory information can improve the visual perception of objects, but it intelligently fails to worsen it when manipulated. So far, we can conclude that we are able to detect inappropriate pairings of image and sound, which enables ignoring distorting information as was the case when altering pitch (cf. [31]). Loudness, however, seems to be more tightly intertwined with visual size as its manipulation produces robust changes in size perception. We speculate that different acoustic dimensions have shown to be unequally reliable cues for size, such that the degree to which their information is taken into account varies.

Further research is needed to pursue this hypothesis. For instance, one could think about the application of more complex experimental settings, in which sounds are manipulated online, thus providing closed-loop feedback, similar to the setup employed by Zampini and Spence [32] in their study on auditory cues to the freshness of potato chips. This way, sound manipulation might be less noticeable by the participants. It would also be interesting to replicate Control Experiment 2 with even stronger distortions of pitch to explore the potential limits of multisensory suppression.

In conclusion, when multisensory information (vision and sound) was available to estimate object length, vision dominated all subtle sound cues (as present when sound track and image were swapped). However, accuracy was improved by multimodal, compared to unimodal, presentation of objects. Brute variations of loudness—in contrast—did sway the length judgments, the louder the longer. Equally, brute pitch manipulations had no such effect.

## Figures and Tables

**Table 1:** Results of RM-ANOVAs (7 Visual Length × 7 Auditory Length × 3 Sound Level).

Factors	*F*	df	*p*	η^2^
	**Absolute Estimates**			
Visual Length	370.12	1.8, 51.53	<0.001 **	0.91
Auditory Length	1.86	6, 210	0.089	0.05
Sound Level	13.32	2, 70	<0.001 **	0.28
Visual Length * Auditory Length	2.98	36, 1260	0.001 **	0.08
Visual Length * Sound Level	2.28	7.69, 269.24	0.024 *	0.06
Auditory Length * Sound Level	1.86	8, 280.1	0.067	0.05
	**Percentage Deviation**			
Visual Length	34.41	1.3, 45.07	<0.001 **	0.5
Auditory Length	1.07	6, 210	0.379	0.03
Sound Level	7.33	2, 70	0.001 **	0.17
Visual Length * Auditory Length	1.9	36, 1260	0.001 **	0.05
Visual Length * Sound Level	1.19	6.21, 217.3	0.29	0.03
Auditory Length * Sound Level	1.86	6.53, 228.53	0.083	0.05

Asterisks indicate significant main effects (* = *p* < 0.05, ** = *p* < 0.01).

## Supplementary Materials

Supplementary materials at https://www.mdpi.com/2411-5150/3/4/57/s1. Datasets of all three experiments are available as SPSS data files in supplementary materials.

## References

[1] Thomas G.J. (1941). Experimental study of the influence of vision on sound localization. J. Exp. Psychol..

[2] Radeau M., Bertelson P. (1987). Auditory-visual interaction and the timing of inputs: Thomas (1941) revisited. Psychol. Res..

[3] Alais D., Burr D. (2004). The ventriloquist effect results from near-optimal bimodal integration. Curr. Biol..

[4] Sekuler R., Sekuler A.B., Lau R. (1997). Sound alters visual motion perception. Nature.

[5] Takeshima Y., Gyoba J. (2013). Changing pitch of sounds alters perceived visual motion trajectory. Multisens. Res..

[6] Klatzky R.L., Pai D.K., Krotkov E.P. (2000). Perception of material from contact sounds. Presence Teleoperators Virtual Environ..

[7] Kunkler-Peck A.J., Turvey M.T. (2000). Hearing shape. J. Exp. Psychol. Hum..

[8] Lakatos S., McAdams S., Caussé R. (1997). The representation of auditory source characteristics: Simple geometric form. Percept. Psychophys..

[9] Lutfi R.A., Liu C.-J., Stoelinga C. (2008). Level dominance in sound source identification. J. Acoust. Soc. Am..

[10] Evans K.K., Treisman A. (2010). Natural cross-modal mappings between visual and auditory features. J. Vis..

[11] Gallace A., Spence C. (2006). Multisensory synesthetic interactions in the speeded classification of visual size. Percept. Psychophys..

[12] Mondloch C.J., Maurer D. (2004). Do small white balls squeak? Pitch-object correspondences in young children. Cogn. Affect. Behav. Neurosci..

[13] Takeshima Y., Gyoba J. (2013). High-intensity sound increases the size of visually perceived objects. Atten. Percept. Psychophys..

[14] Houben M.M.J., Kohlrausch A., Hermes D.J. (2004). Perception of the size and speed of rolling balls by sound. Speech Commun..

[15] Patterson R.D., Smith D.R., Dinther R., Walters T.C. (2008). Size information in the production and perception of communication sounds. Auditory Perception of Sound Sources.

[16] Carello C., Anderson K.L., Kunkler-Peck A.J. (1998). Perception of object length by sound. Psychol. Sci..

[17] Carello C., Wagman J.B., Turvey M.T., Anderson J.D., Fisher Anderson B. (2005). Acoustic specification of object properties. Moving Image Theory: Ecological Considerations.

[18] Gaver W.W. (1993). What in the world do we hear? An ecological approach to auditory event perception. Ecol. Psychol..

[19] Gibson J.J. (1978). The ecological approach to the visual perception of pictures. Leonardo.

[20] Grassi M. (2005). Do we hear size or sound? Balls dropped on plates. Percept. Psychophys..

[21] Charpentier A. (1981). Analyse expérimentale de quelques éléments de la sensation de poids. Arch. Physiol. Norm. Pathol..

[22] Murray D.J., Ellis R.R., Bandomir C.A., Ross H.E. (1999). Charpentier (1891) on the size-weight illusion. Percept. Psychophys..

[23] Walker P., Scallon G., Francis B. (2017). Cross-sensory correspondences: Heaviness is dark and low-pitched. Perception.

[24] Walker L., Walker P., Francis B. (2012). A common scheme for cross-sensory correspondences across stimulus domains. Perception.

[25] Lipscomb S.D., Kim E.M. Perceived match between visual parameters and auditory correlates: An experimental multimedia investigation. Proceedings of the 8th International Conference on Music Perception and Cognition.

[26] Odegaard B., Wozny D.R., Shams L. (2015). Biases in visual, auditory, and audiovisual perception of space. PLoS Comput. Biol..

[27] McGurk H., Macdonald J. (1976). Hearing lips and seeing voices. Nature.

[28] Colavita F.B. (1974). Human sensory dominance. Percept. Psychophys..

[29] van Damme S., Crombez G., Spence C. (2009). Is visual dominance modulated by the threat value of visual and auditory stimuli?. Exp. Brain Res..

[30] Tsay C.-J. (2013). Sight over sound in the judgment of music performance. Proc. Natl. Acad. Sci. USA.

[31] Grassi M., Pastore M., Lemaitre G. (2013). Looking at the world with your ears: How do we get the size of an object from its sound?. Acta Psychol..

[32] Zampini M., Spence C. (2004). The role of auditory cues in modulating the perceived crispness and staleness of potato chips. J. Sens. Stud..

